# Physiological effects of short acute UVB treatments in *Chenopodium quinoa* Willd

**DOI:** 10.1038/s41598-017-18710-2

**Published:** 2018-01-10

**Authors:** Thais Huarancca Reyes, Andrea Scartazza, Antonella Castagna, Eric G. Cosio, Annamaria Ranieri, Lorenzo Guglielminetti

**Affiliations:** 1 Department of Agriculture, Food and Environment, University of Pisa, Via del Borghetto 80, 56124 Pisa, Italy; 2 Institute of Agro-environmental and Forest Biology, National Research Council, Via Salaria km 29,300, Monterotondo Scalo (RM), 00016 Italy; 3 Sección Química, Pontificia Universidad Católica del Perú, Av. Universitaria 1801, Lima, Lima 32 Peru; 4 Interdepartmental Research Center “Nutraceuticals and Food for Health”, University of Pisa, Via del Borghetto 80, 56124 Pisa, Italy

## Abstract

Increased ultraviolet B (UVB) radiation due to global change can affect plant growth and metabolism. Here, we evaluated the capacity of quinoa to resist under short acute UVB irradiation. Quinoa was daily exposed for 30 or 60 min to 1.69 W m^−2^ UVB. The results showed that 30 min exposure in 9 d-course did not cause severe alterations on photosynthetic pigments and flavonoids, but a significant increase of antioxidant capacity was observed. Otherwise, 60 min UVB in 5 d-course reduced almost all these parameters except for an increase in the de-epoxidation of xanthophyll cycle pigments and led to the death of the plants. Further studies of gas exchange and fluorescence measurements showed that 30 min UVB dramatically decrease stomatal conductance, probably associated to reactive oxygen species (ROS) production. Inhibition of photosynthetic electron transport was also observed, which could be a response to reduce ROS. Otherwise, irreversible damage to the photosynthetic apparatus was found with 60 min UVB probably due to severe ROS overproduction that decompensates the redox balance inducing UVB non-specific signaling. Moreover, 60 min UVB compromised Rubisco carboxylase activity and photosynthetic electron transport. Overall, these data suggest that quinoa modulates different response mechanisms depending on the UVB irradiation dosage.

## Introduction

Solar radiation is the primary source of energy for metabolism in plants and for the regulation of their growth and development. Depletion of the stratospheric ozone layer, a component of global climate change, has prompted studies on the effect of enhanced ultraviolet B (UVB) radiation on growth and yield of agriculture crops^1,2^. Although UVB only represents a fraction of the solar spectrum, its high energy has a substantial impact on living organisms^3^. It has been reported that depending on the flow rate, duration and interaction with other environmental factors, the UVB radiation can induce photomorphogenic and stress responses which are not mutually exclusive. Thus, exposure of plants to low doses of UVB can induce photomorphogenic effects via the UVB RESISTANCE LOCUS 8 (UVR8) photoreceptor^4^. However, high UVB doses can induce the production of reactive oxygen species (ROS), reduction of photosynthesis and damages to cell membranes, proteins and DNA^5–7^.

In natural environments, the intensity of UVB radiation depends on the season, latitude, altitude and clouds, leading to wide temporal and spatial dynamics. Intense UVB radiation is a common characteristic of the Andean region in South America due to the high altitude and thin ozone column^8^. Moreover, the Andes have long periods of drought and freezing temperatures during the dry season; however, we can find Andean crops which have evolved under these extreme conditions in what nowadays would be considered marginal lands^9^. In this line, species grown at highland regions seems to be better adapted to high UVB doses than those grown at lowland regions^10^. Consequently, understanding the strategies involved in UVB tolerance by these plants is important to reveal the detailed response mechanisms allowing adaptation to high UVB and their associated signaling pathways.

Quinoa (*Chenopodium quinoa* Willd.) is a grain crop from the Andean region which has received worldwide interest in the past two decades due to its high protein and well-balanced amino acid content^11,12^. In addition, it has been reported that quinoa can grow under high salinity levels^13^, drought^14^ and high UV radiation^15^. However, the physiological mechanisms behind its abiotic stress tolerance are still unclear, especially those related to its responses to UVB. The aim of this study was to understand the physiological responses of quinoa to different doses of acute UVB irradiance (1.69 W m^−2^). Plants were irradiated daily with UVB for 30 or 60 min, and the physiological responses reflected in their capacity to maintain photosynthetic activity were evaluated by: (i) changes in fluorescence parameters of photosystem II (PSII), (ii) gas exchanges, (iii) changes in photosynthetic pigments content, (iv) changes in non-enzymatic antioxidant capacity and (v) protein degradation.

## Results

### Chlorophyll fluorescence parameters

**Figure 1:**
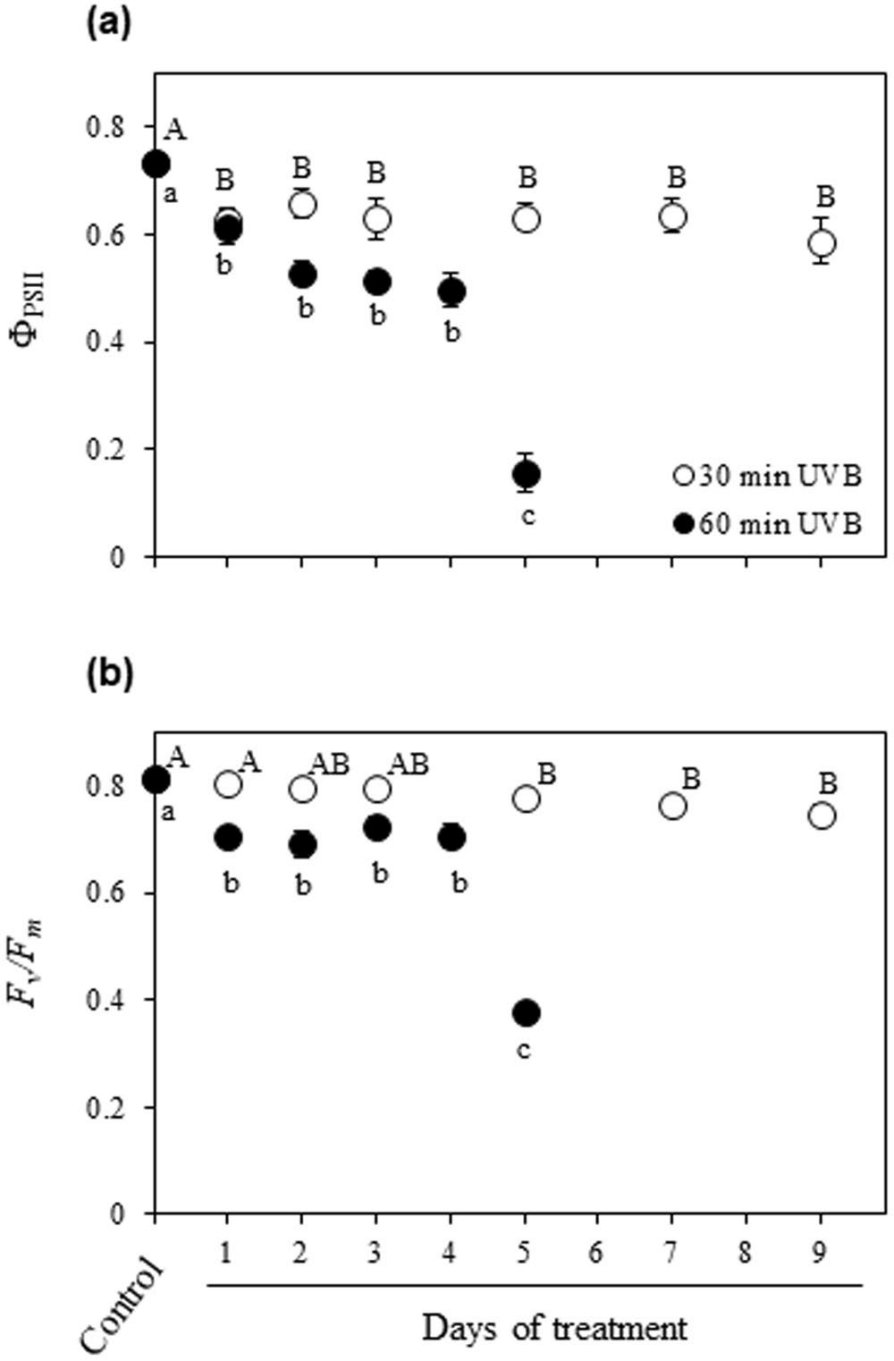
Effects of UVB and treatment period on the maximum (*F*_*v*_ */F*_*m*_) and actual (Φ_PSII_) efficiency of PSII photochemistry. Quinoa plants were exposed to 100 µmol m^−2^ s^−1^ photosynthetic photon flux density (PPFD) and 1.69 W m^−2^ UVB. Φ_PSII_ (**a**) and *F*_*v*_ */F*_*m*_ (**b**) were evaluated in leaves that daily applied for 30 or 60 min UVB in a time course. Control plants only received PPFD and their temporal evolution did not show any statistical differences as shown in Supplementary Table S1. Error bars represent the standard error of the mean (n = 3). The different letters indicate significant differences between means tested using one-way ANOVA and Tukey tests (*P* < 0.05).

The two-way ANOVA analysis revealed a significant effect of UVB treatment (*P* < 0.001) and time (*P* < 0.001), as well as the interaction treatment x time (*P* < 0.001) on different tested parameters. Exposure to 30 min UVB for 1 d showed a significant decrease of the actual photon yield of PSII photochemistry (Φ_PSII_) in comparison with that of control, and then Φ_PSII_ remained stable for a period of 9 d of treatment (DOT). Conversely, plants exposed to 60 min UVB showed a first decline of Φ_PSII_ at 1 DOT with a further abrupt decrease at 5 DOT in comparison to that of control (Fig. 1a). Minor effects on maximal PSII photochemical efficiency (*F*_*v*_ */F*_*m*_) were observed in plants exposed to 30 min UVB in the time course up to 3 DOT, followed by a slight but significant decrease from 5 to 9 DOT in comparison to that of control. Plants exposed to 60 min UVB showed a significant decrease of *F*_*v*_ */F*_*m*_ at 1 DOT in comparison to that of control, followed by a dramatic decrease at 5 DOT with strong damage in the leaves of plants (Fig. 1b).

### Photosynthetic pigments

**Figure 2:**
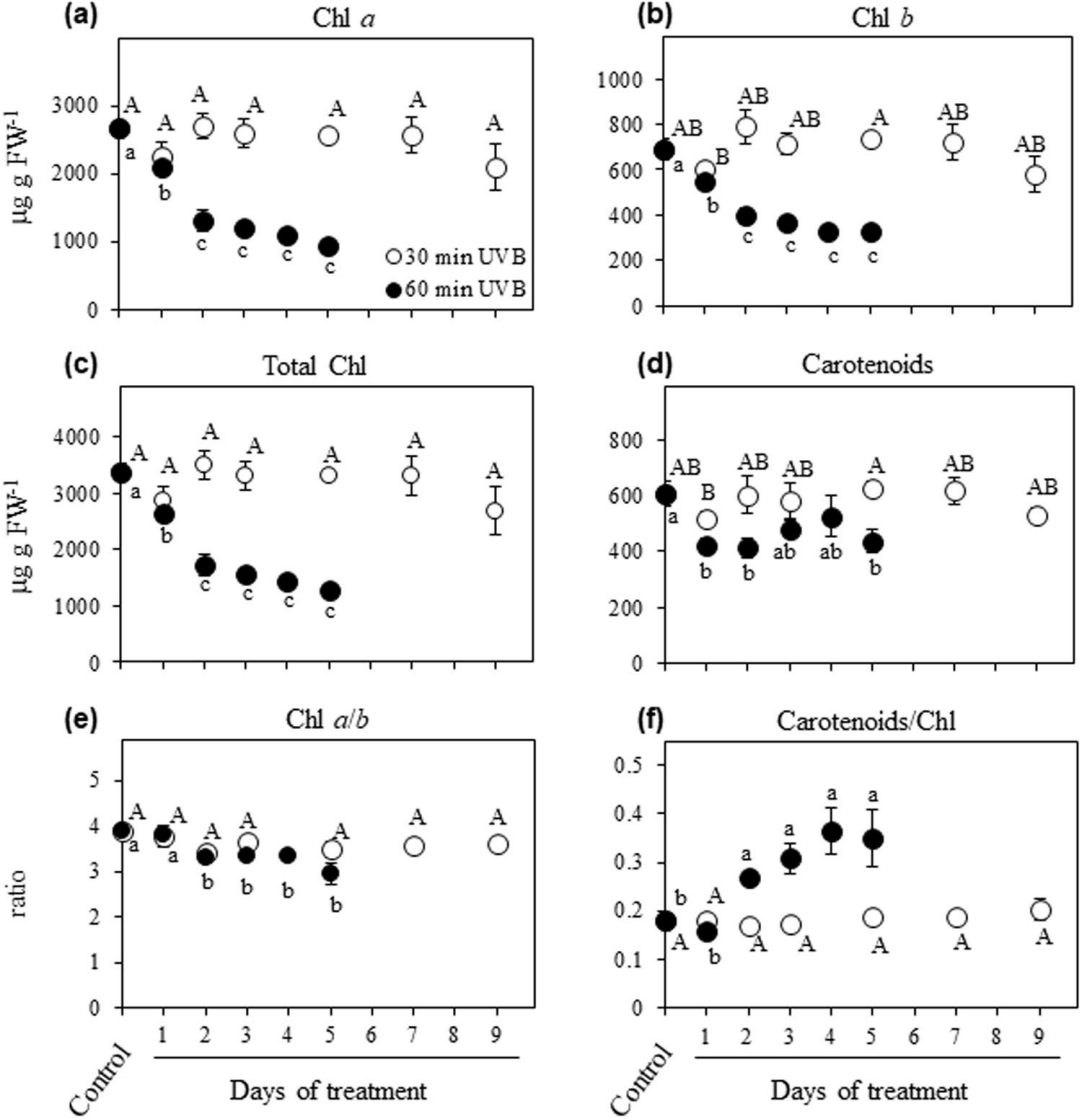
Effects of UVB and treatment period on the photosynthetic pigment contents and some pigment ratios. Quinoa plants were exposed to 100 µmol m^−2^ s^−1^ photosynthetic photon flux density (PPFD) and 1.69 W m^−2^ UVB. Chl *a* (**a**), Chl *b* (**b**), total chlorophyll (**c**), carotenoids (**d**), and the ratios of Chl *a*/*b* (**e**) and carotenoids to total chlorophyll (**f**) were evaluated in leaves that daily received 30 or 60 min UVB in a time course. Control plants only received PPFD and their temporal evolution did not show any statistical differences as shown in Supplementary Table S1. Error bars represent the standard error of the mean (n = 3). The different letters indicate significant differences between means tested using one-way ANOVA and Tukey tests (*P* < 0.05). FW, fresh weight.

The two-way ANOVA analysis revealed a significant effect of UVB treatment (*P* < 0.001) and time (*P* < 0.001), as well as the interaction treatment x time (*P* < 0.001) on different tested parameters. 30 min UVB irradiation did not show a strong effect on chlorophyll (Chl) *a*, Chl *b* and total Chl in the time course up to 9 DOT in comparison with the control, while 60 min UVB greatly depressed leaf Chl concentration in comparison with control plants (Fig. 2a–c). Total carotenoid content was not sensitive to 30 min UVB radiation during the time course, while in plants exposed to 60 min UVB carotenoids decreased at 1 DOT in comparison with the control and then remained unchanged up to 5 DOT (Fig. 2d). Chl *a*/*b* ratio showed no differences when plants were exposed 30 min UVB from 1 to 9 DOT respect to the control plants, while in the case of 60 min UVB the ratio showed a slight decrease in the time course (Fig. 2e). Interestingly, plants exposed to 30 min UVB in the time course maintained same carotenoid to total Chl ratio as the control, while 60 min UVB irradiation led to a gradual increase of the ratio during the time course up to the maximum at 4 DOT (Fig. 2f).

### Xanthophyll cycle pigments

**Figure 3:**
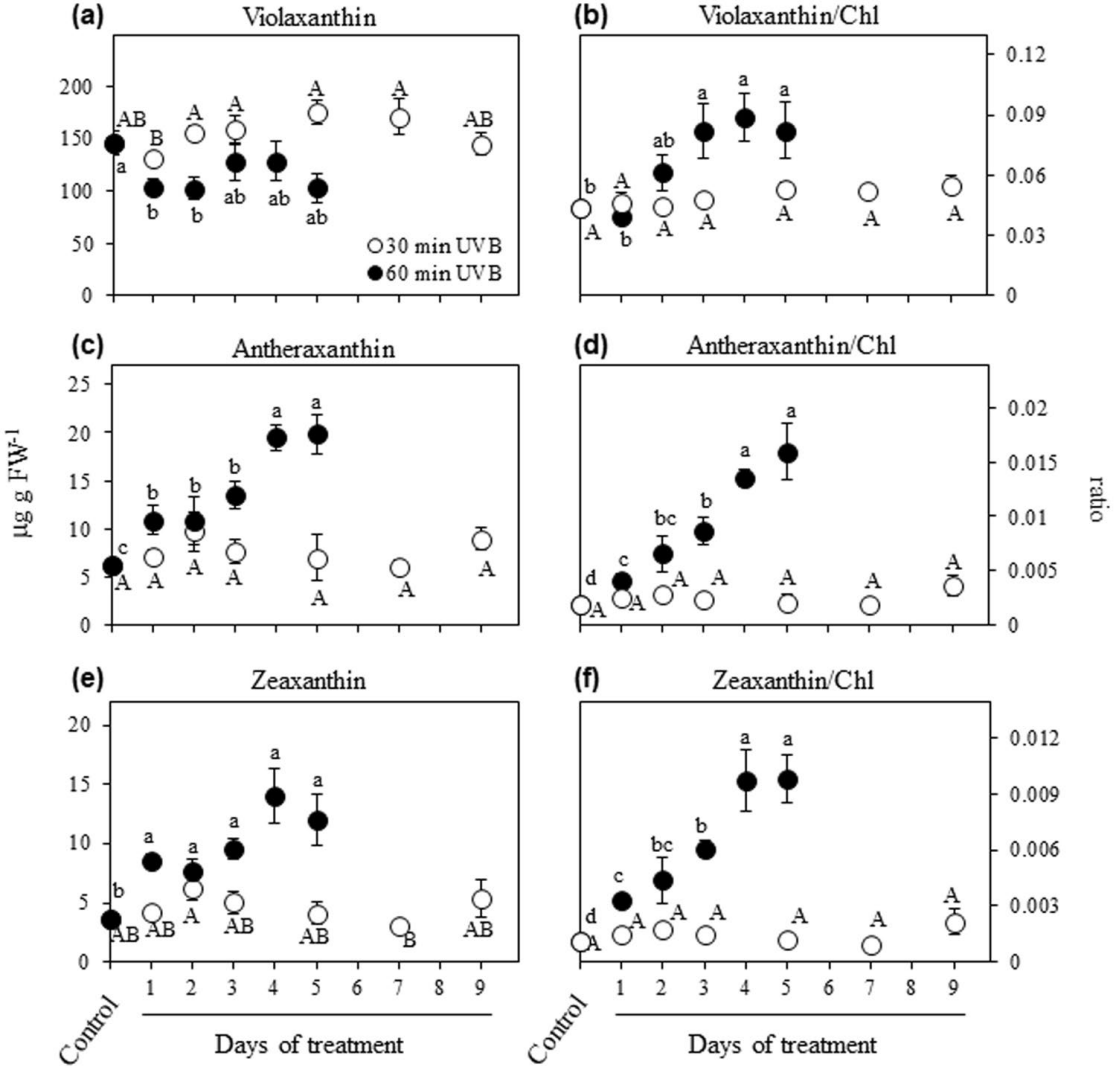
Effects of UVB and treatment period on the xanthophyll cycle pigment contents and some pigment ratios. Quinoa plants were exposed to 100 µmol m^−2^ s^−1^ photosynthetic photon flux density (PPFD) and 1.69 W m^−2^ UVB. Violaxanthin (**a**), antheraxanthin (**c**), zeaxanthin (**e**), and the ratios between each pigment respect to the total chlorophyll (**b**,**d**,**f**) were evaluated in leaves that daily received 30 or 60 min UVB in a time course. Control plants only received PPFD and their temporal evolution did not show any statistical differences as shown in Supplementary Table S1. Error bars represent the standard error of the mean (n = 3). The different letters indicate significant differences between means tested using one-way ANOVA and Tukey tests (*P* < 0.05). FW, fresh weight.

**Figure 4:**
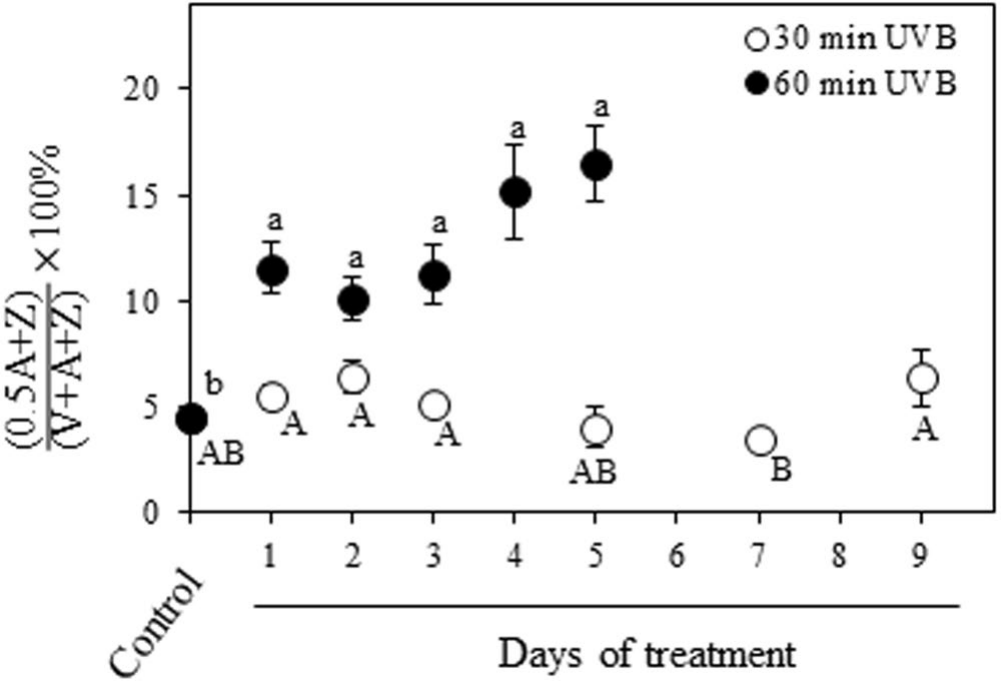
Effects of UVB and treatment period on the de-epoxidation state of xanthophyll cycle pigments. Quinoa plants were exposed to 100 µmol m^−2^ s^−1^ photosynthetic photon flux density (PPFD) and 1.69 W m^−2^ UVB. Treated plants daily received 30 or 60 min UVB in a time course. Control plants only received PPFD and their temporal evolution did not show any statistical differences as shown in Supplementary Table S1. Error bars represent the standard error of the mean (n = 3). The different letters indicate significant differences between means tested using one-way ANOVA and Tukey tests (*P* < 0.05). A, antheraxanthin. Z, zeaxanthin. V, violaxanthin.

The two-way ANOVA analysis revealed a significant effect of UVB treatment (*P* < 0.001) and time (*P* < 0.001), as well as the interaction treatment x time (*P* < 0.001) on different tested parameters. In plants exposed to 30 min UVB radiation, the xanthophyll cycle pigments did not show strong differences during the time course in comparison to the control (Fig. 3a,c,e). This result was consistent with the de-epoxidation of violaxanthin to antheraxanthin and zeaxanthin which was not strongly affected in leaves at the same conditions (Fig. 4). Interestingly, 60 min UVB dose radiation showed a gradual increase in the content of antheraxanthin, zeaxanthin and de-epoxidation state during the course of the experiment in comparison to that of the control (Figs 3c,e and 4), while the content of violaxanthin slightly decreased at 1 DOT, and then fluctuated close to the control level (Fig. 3a). The ratios of violaxanthin, antheraxanthin and zeaxanthin pools relative to total Chl remained similar to control level during the time course with 30 min UVB dose radiation, while they gradually increased when the UVB dose was extended to 60 min (Fig. 3b,d,f).

### Flavonoids and antioxidants content

**Figure 5:**
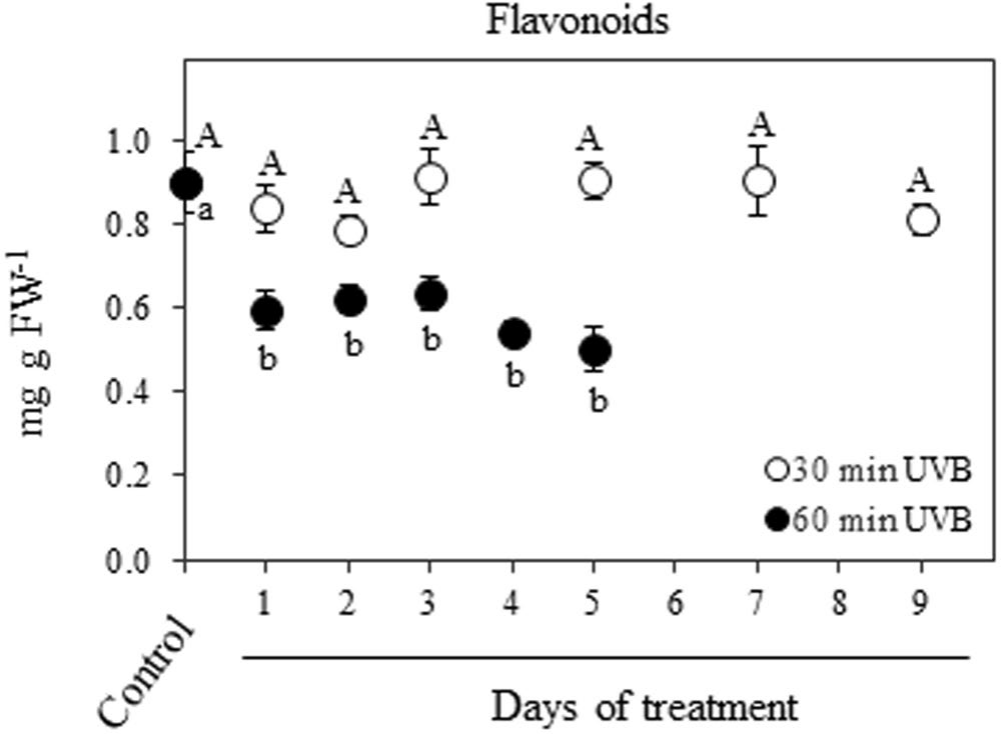
Effects of UVB and treatment period on flavonoids content. Quinoa plants were exposed to 100 µmol m^−2^ s^−1^ photosynthetic photon flux density (PPFD) and 1.69 W m^−2^ UVB. Treated plants daily received 30 or 60 min UVB in a time course. Control plants only received PPFD and their temporal evolution did not show any statistical differences as shown in Supplementary Table S1. Error bars represent the standard error of the mean (n = 3). The different letters indicate significant differences between means tested using one-way ANOVA and Tukey tests (*P* < 0.05). FW, fresh weight.

**Figure 6:**
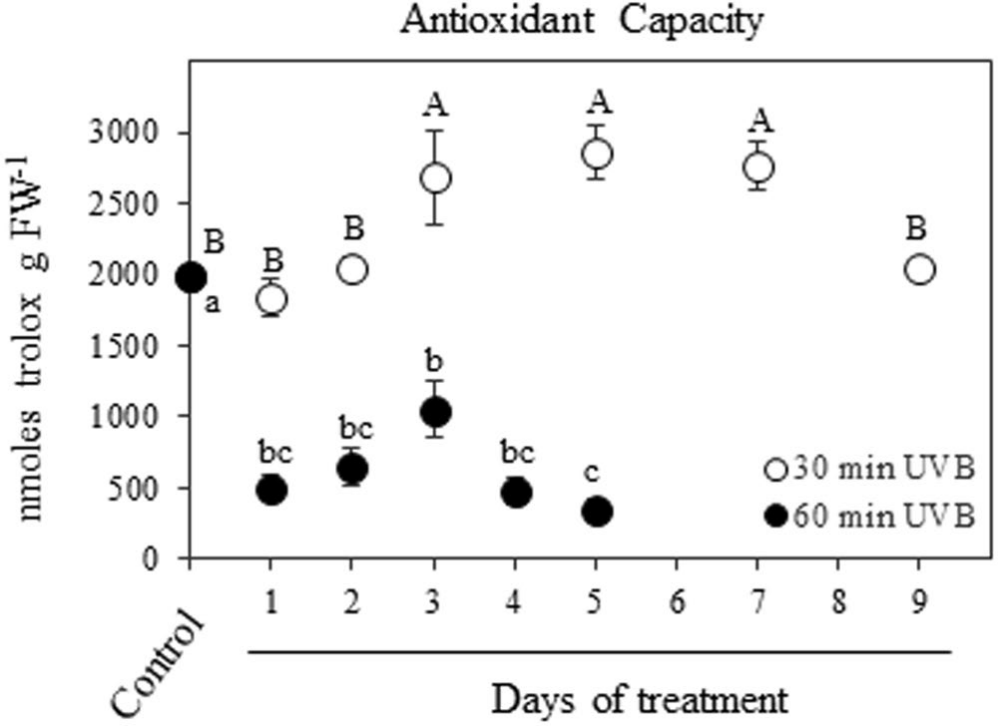
Effects of UVB and treatment period on the non-enzymatic antioxidant capacity. Quinoa plants were exposed to 100 µmol m^−2^ s^−1^ photosynthetic photon flux density (PPFD) and 1.69 W m^−2^ UVB. Treated plants daily received 30 or 60 min UVB in a time course. Control plants only received PPFD and their temporal evolution did not show any statistical differences as shown in Supplementary Table S1. Error bars represent the standard error of the mean (n = 3). The different letters indicate significant differences between means tested using one-way ANOVA and Tukey tests (*P* < 0.05). FW, fresh weight.

The two-way ANOVA analysis revealed a significant effect of UVB treatment (*P* < 0.001) and time (*P* < 0.001), as well as the interaction treatment x time (*P* < 0.001) on different tested parameters. Quinoa leaves exposed to 30 min UVB dose radiation maintained similar content of total flavonoids during the time course respect to the control, while plants receiving 60 min UVB at 1 DOT showed a significant decrease in flavonoid content (approximately 30%) in comparison to the control which then remained constant up to 5 DOT (Fig. 5). The antioxidant capacity of quinoa exposed to 30 min UVB showed transient increase respect to the control level after 3 DOT, the value being maintained up to 7 DOT and followed by a decrease at 9 DOT to the control level (Fig. 6). On the contrary, quinoa leaves receiving 60 min UVB showed a 75% reduction in antioxidant activity at 1 DOT in comparison to the control, and this reduced level remained until the end of the experiment (5 DOT; Fig. 6).

### The effect of different UVB doses on the photosynthetic parameters

**Figure 7:**
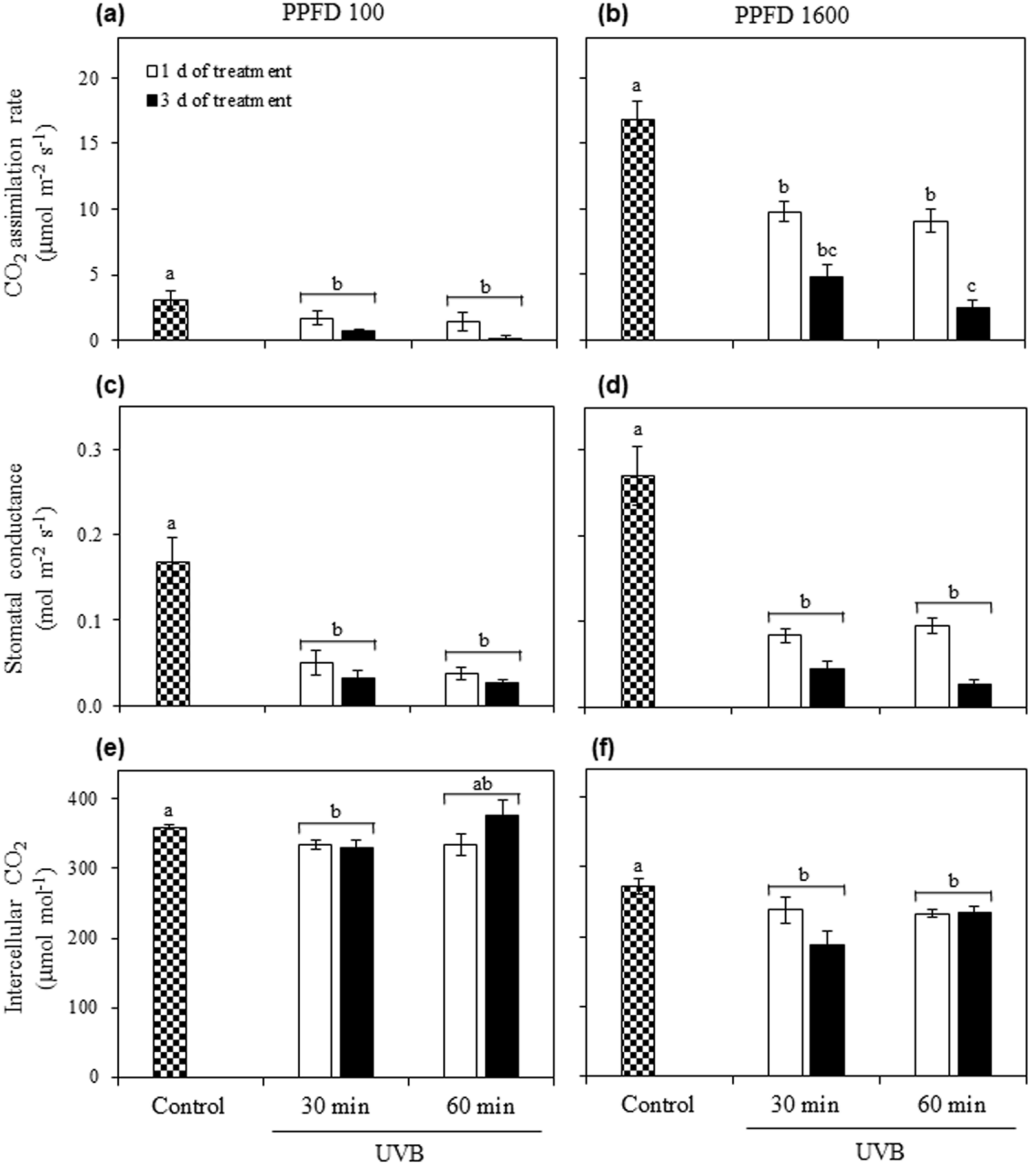
Leaf gas exchanges of UVB-irradiated quinoa plants in the three days of treatment. Quinoa was exposed to 100 µmol m^−2^ s^−1^ photosynthetic photon flux density (PPFD) and 1.69 W m^−2^ UVB. Treated plants daily received 30 or 60 min UVB for 1 or 3 days. Control plants only received PPFD and their temporal evolution did not show any statistical differences as shown in Supplementary Table S2. Parameters of CO_2_ assimilation rate (**a**,**b**), stomatal conductance (**c**,**d**) and intercellular CO_2_ concentration (**e**,**f**) were evaluated at 100 µmol m^−2^ s^−1^ PPFD (**a**,**c**,**e**) and 1600 µmol m^−2^ s^−1^ PPFD (**b**,**d**,**f**). Error bars represent the standard error of the mean (n = 3). The different letters indicate significant differences between means tested using one-way ANOVA and Tukey tests (*P* < 0.05).

Gas exchanges were evaluated at 1 and 3 DOT (selected according to the results from the PSII photochemical efficiency experiment) in comparison to the control at growth light condition (Fig. 7a,c,e) and at saturating light (Fig. 7b,d,f). The two-way ANOVA analysis revealed a significant effect of UVB treatment (*P* < 0.01) on CO_2_ assimilation rates (*A*) at PPFD 100, with a reduced *A* in quinoa leaves irradiated with 30 or 60 min UVB in comparison to the control, without significant effects of time of treatment and UVB doses (Fig. 7a). Increasing PPFD to 1600 µmol m^−2^ s^−1^, *A* was significantly reduced by the treatment (*P* < 0.0001), with a significant effect of the time of treatment (1 or 3 DOT; *P* < 0.01) and of the interaction between treatment and time (*P* < 0.05). At PPFD 1600, *A* was significantly reduced in comparison to the control at 1 DOT in both UVB treatments, with a further reduction in 60 min UVB treatment at 3 DOT (Fig. 7b). Evaluation of stomatal conductance (*g*_*s*_) in leaves exposed to 30 or 60 min UVB resulted in a significant decrease respect to the control under growth light condition (*P* < 0.0001), without significant differences between 1 and 3 DOT, and between different UVB radiation doses (Fig. 7c). At PPFD 1600, *g*_*s*_ showed a similar pattern to that of PPFD 100 (Fig. 7d). Intercellular CO_2_ concentration (*C*_*i*_) in quinoa leaves at PPFD 100 was significantly affected by the treatments (*P* < 0.05) with a significant reduction at 30 min UVB treatment in comparison with control, without significant differences between 1 and 3 DOT (Fig. 7e). However, at PPFD 1600, *C*_*i*_ was significantly reduced by both UVB treatments (*P* < 0.0001), without significant effect of the time (Fig. 7f).

**Figure 8:**
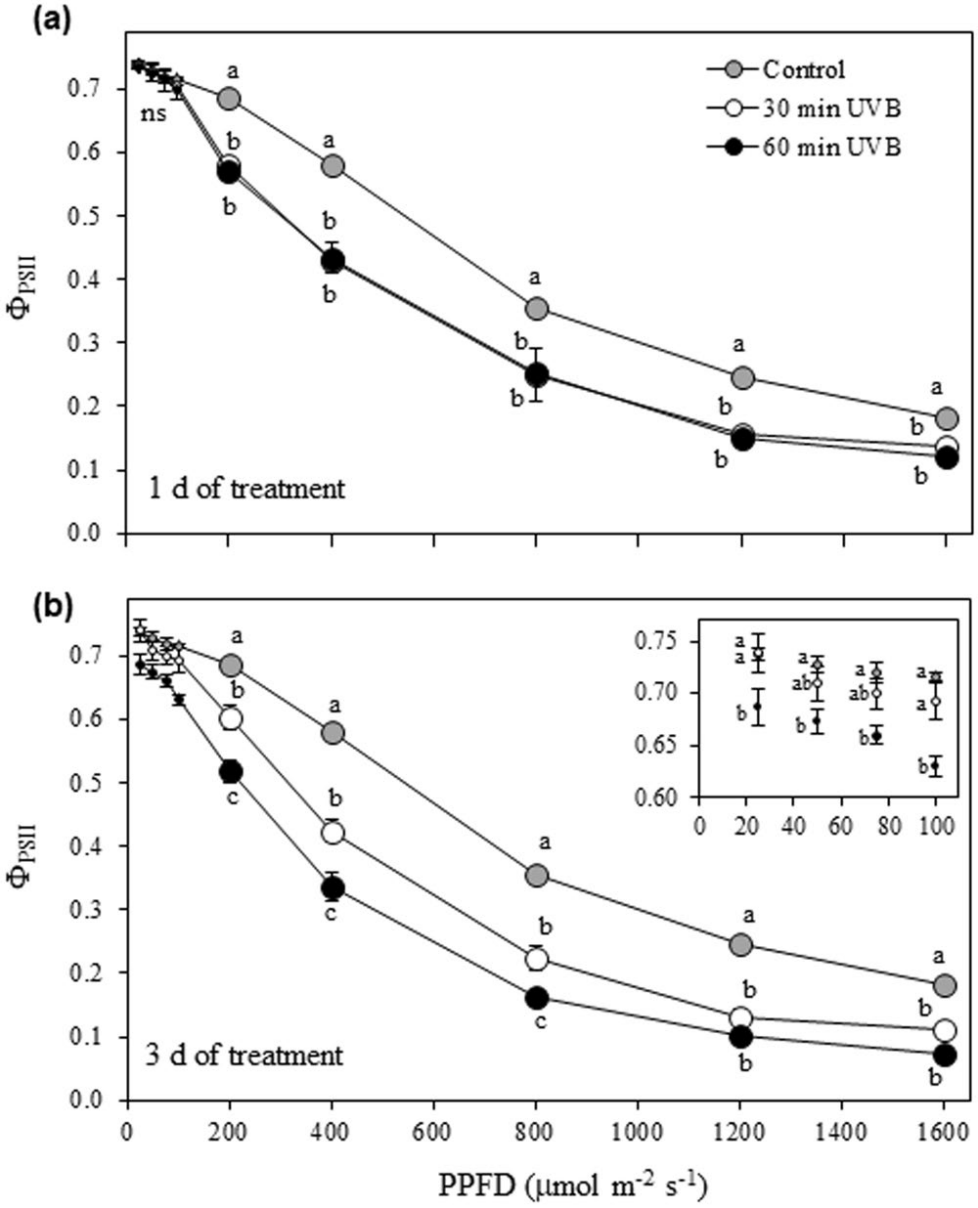
Light-response curves of the actual (Φ_PSII_) efficiency of PSII photochemistry in UVB irradiated quinoa plants. Quinoa was exposed to 100 µmol m^−2^ s^−1^ photosynthetic photon flux density (PPFD) and 1.69 W m^−2^ UVB. Treated plants daily received 30 or 60 min UVB for 1 (**a**) or 3 (**b**) days. Control plants only received PPFD. Error bars represent the standard error of the mean (n = 3). The different letters indicate significant differences between means at determined PPFD tested using one-way ANOVA and Tukey tests (*P* < 0.05). ns, not significant.

The three-way ANOVA analysis of Φ_PSII_ in quinoa leaves irradiated with 30 or 60 min UVB revealed a significant effect of PPFD (*P* < 0.0001), treatment (*P* < 0.0001) and time (*P* < 0.0001), as well as the interactions PPFD x treatment (*P* < 0.0001) and treatment x time (*P* < 0.0001). A significant decrease of Φ_PSII_ respect to the control was observed when PPFD ≥200 µmol m^−2^ s^−1^ at 1 DOT, without significant differences between the UVB doses (Fig. 8a). At 3 DOT, 30 min UVB irradiated quinoa leaves showed similar Φ_PSII_ as the control, while 60 min UVB were significantly lower when PPFD ≤100 µmol m^−2^ s^−1^. Differently, Φ_PSII_ in leaves irradiated 30 min UVB were significantly lower than the control but higher than 60 min UVB dose when PPFD was increased up to 800 µmol m^−2^ s^−1^ (Fig. 8b).

**Figure 9:**
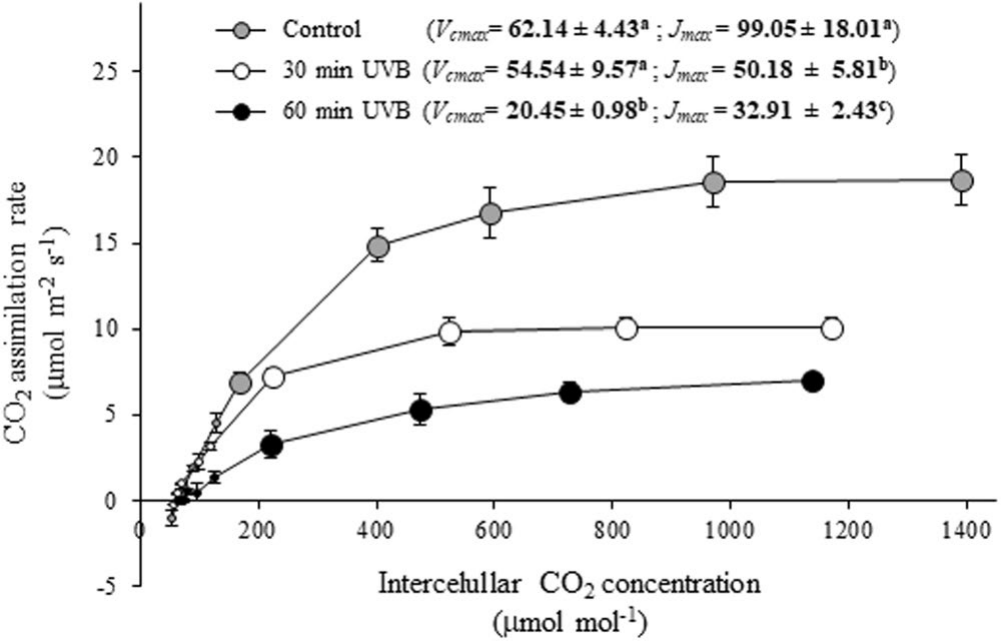
Response curves of CO_2_ assimilation rate *versus* intercellular CO_2_ concentration (*A*/*C*_*i*_ curves) in UVB irradiated quinoa plants. Quinoa was exposed to 100 µmol m^−2^ s^−1^ photosynthetic photon flux density (PPFD) and 1.69 W m^−2^ UVB. Treated plants daily received 30 or 60 min UVB doses for 3 days. Control plants only received PPFD. The *A*/*C*_*i*_ curves were carried out at saturating light intensity (1600 μmol m^−2^ s^−1^). Values of maximal carboxylation rate (*V*_*cmax*_) and maximal light-driven electron transport rate (*J*_*max*_) are reported. Error bars represent the standard error of the mean (n = 3). The different superscript letters indicate significant differences between means tested using one-way ANOVA and Tukey tests (*P* < 0.05).

Moreover, the response curves of CO_2_ assimilation rate to intercellular CO_2_ concentration (*A*/*C*_*i*_) at 3 DOT showed that the maximum rate of Rubisco carboxylase activity (*V*_*cmax*_) was similar between leaves exposed to 30 min UVB radiation and the control, while was significantly lower in leaves irradiated with 60 min UVB. The maximum rate of photosynthetic electron transport (*J*_*max*_) was significantly lower in leaves irradiated 30 min UVB respect to the control, and 60 min UVB irradiated leaves showed even much lower *J*_*max*_ than 30 min UVB dose (Fig. 9).

### Recovery after different UVB doses

Quinoa plants irradiated 30 min with UVB could recover their photochemical efficiency even after 9 d of treatment without significant differences respect to the contemporary UVB-untreated plants (control) (Table 1). On the contrary, leaves exposed to 60 min UVB radiation showed significantly lower Φ_PSII_ and *F*_*v*_ */F*_*m*_ values than the control for exposure periods from 1–3 d and 3 d, respectively. Moreover, the damages in 60 min UVB irradiated quinoa leaves for 4 or 5 d proved irreversible and these plants finally died.

**Table 1:** Changes in the actual (Φ_PSII_) and maximum (*F*_*v*_ */F*_*m*_) efficiency of PSII photochemistry after 7 days of recovery in untreated and UVB treated quinoa plants.

Control		30 min UVB			60 min UVB		
Φ_PSII_	*F*_*v*_ */F*_*m*_	*DOT*	Φ_PSII_	*F*_*v*_ */F*_*m*_	*DOT*	Φ_PSII_	*F*_*v*_ */F*_*m*_
0.73 ± 0.01^a^	0.82 ± 0.00^a^	***1***	0.72 ± 0.02^a^	0.81 ± 0.01^a^	***1***	0.69 ± 0.01^b^	0.81 ± 0.01^a^
		***2***	0.74 ± 0.00^a^	0.81 ± 0.01^a^	***2***	0.64 ± 0.01^b^	0.78 ± 0.02^a^
		***3***	0.73 ± 0.01^a^	0.81 ± 0.01^a^	***3***	0.54 ± 0.06^b^	0.71 ± 0.01^b^
		***5***	0.72 ± 0.01^a^	0.81 ± 0.00^a^	***4***	nd	nd
		***7***	0.72 ± 0.01^a^	0.80 ± 0.01^a^	***5***	nd	nd
		***9***	0.67 ± 0.03^a^	0.75 ± 0.0^a^			

Prior to recovery, plants had been exposed to either 100 µmol m^−2^ s^−1^ photosynthetic photon flux density (PPFD) or 1.69 W m^−2^ UVB. UVB had been daily applied for 30 min in 9 days course or for 60 min in 5 days course. Control plants had only received PPFD. Each value represents the mean ± standard error (n = 3). Different superscript letters indicate significant differences between means tested using one-way ANOVA and Tukey tests (*P* < 0.05). nd, not detected. DOT, days of treatment.

## Discussion

In this study, we tested the capacity of quinoa plants to resist under short acute UVB irradiance (1.69 W m^−2^) based on the knowledge that this species is well-adapted to relatively high UVB^11^. Our results suggest that quinoa modulates different mechanisms of response depending on the UVB irradiation dosage, which were reflected on the capacity to maintain its photosynthetic activity.

Exposure to 30 min UVB for 3 d (9.12 kJ m^−2^ dose) did not affect Φ_PSII_ values at growth light condition (PPFD 100 µmol m^−2^ s^−1^). However, exclusion of the light limiting factor by increasing PPFD to 1600 µmol m^−2^ s^−1^ revealed a reduction in photosynthesis associated with a limitation of gas exchange. In fact, it has been demonstrated that among the known physiological responses to UVB radiation are a reduction in photosynthetic activity mainly related to the degradation of PSII proteins, destruction of chlorophyll and carotenoids, reduced Rubisco activity and effects on stomatal functions^6^. Interestingly, quinoa plants at UVB irradiation dose of 9.2 kJ m^−2^ did not show any effect on the photosynthetic pigments such as chlorophylls and carotenoids, as reported in a meta-analysis of polar plants^16^. UVB absorbing pigments such as flavonoids were also unaffected; however, the total antioxidant capacity was increased by UVB indicating that ROS were produced. UVB has been recognized as a potential source of oxidative stress producing ROS such as hydroxyl radicals (HO˙), singlet oxygen (^1^O_2_), superoxide (O_2_^−^) and hydrogen peroxide (H_2_O_2_)^5,17^. ROS are considered not only stress-inducing compounds but also signaling molecules that control many different processes in plants such as growth, development and defense pathways^18^. Tossi *et al*.^19^ reported that UVB influences on the stomatal movement via UVR8 signaling, which elicits the production of H_2_O_2_ and nitric oxide (NO) and thus causing stomatal closure in Arabidopsis. Studies performed by Zhu *et al*.^20^ proposed that UVB-induced stomatal closure in Arabidopsis was caused by H_2_O_2_ production and feedback regulation of the cytosolic alkalization in guard cells. Concordantly, a 9.2 kJ m^−2^ UVB dose caused a decrease of CO_2_ assimilation rate related with the strong reduction of stomatal conductance, which may be associated to ROS production via UVB-specific signaling in guard cells. In addition, the respective *A*/*C*_*i*_ curve revealed that *J*_*max*_ was significantly reduced without effect on *V*_*cmax*_, indicating that the carbon fixation pathway was mainly affected by the inhibition of photosynthetic electron transport which could be considered a response to prevent ROS accumulation by UVB^18^. Thus, we speculate that quinoa exposed to 1.69 W m^−2^ for 30 min doses induces UVB-specific signaling, which involves the interaction of several regulators in order to counteract ROS overproduction and avoid damage to the PSII reaction centers as reflected by the unaffected *F*_*v*_ */F*_*m*_ parameter.

On the contrary, exposure to 60 min UVB for 3 d (18.24 kJ m^−2^ dose) revealed a different physiological response on quinoa. The increasing UVB levels strongly reduced Φ_PSII_ and *F*_*v*_ */F*_*m*_ values, indicating an inhibition of photochemistry activity due to detrimental effects on the functioning of PSII. Our results also revealed that the decline in photosynthesis was caused by the decrease of chlorophylls with a significant reduction of Chl *a*/*b* ratio, suggesting that enhanced UVB dose may induce selective degradation pathways of Chl *a* as previously reported^21,22^. Similar UVB-induced inhibition of photosynthesis resulting from chlorophylls degradation was also observed in some cultivars of quinoa^15,23,24^. In addition, enhanced UVB radiation can not only cause the reduction of chlorophylls, but also of carotenoids as reported in several crop species^25,26^. In this study, exposure to higher UVB irradiation (18.24 kJ m^−2^ dose) slightly reduced carotenoids content in comparison to chlorophylls, as reflected by the significant increase of the Carotenoids/Chlorophylls ratio. Interestingly, among carotenoids, our results point to a pronounced increase in xanthophyll cycle pigments with high induction of the de-epoxidation state, where these pigments are involved in thermal dissipation of excitation energy in plants under saturating light conditions^27^. Another mechanism of photoprotection from UVB radiation is the accumulation of UVB absorbing pigments such as flavonoids; however, in our experiment 18.24 kJ m^−2^ UVB dose significantly decreased the concentration of flavonoids. Guidi *et al*.^28^ found that UV radiation increases flavonoid content while inhibiting the biosynthesis and de-epoxidation of xanthophylls, proposing that there is a trade-off between flavonoid and xanthophyll biosynthesis. In this line, it is possible that quinoa could differentially regulate pathways between non-photosynthetic and photosynthetic pigments in response to high UVB flux. Thus, xanthophyll pigments might be relevant in the protection of chloroplasts from UV-induced photo-oxidative damage. Despite the photoprotective role of xanthophyll pigments^29^, we found that the 18.24 kJ m^−2^ UVB dose induced the depression of total non-enzymatic antioxidant capacity, which may be related to severe ROS overproduction and the consequent shift of the redox balance towards oxidative stress^17^. We also observed that the 18.24 kJ m^−2^ UVB dose negatively affected stomatal conductance that, together with the non-stomatal effect, led to a reduced assimilation rate, much lower than that of 9.12 kJ m^−2^ UVB dose. Altogether these effects resulted in a decrease in the carboxylation capacity reflected on the higher reduction of *V*_*cmax*_ and *J*_*max*_ in comparison with control or the 9.12 kJ m^−2^ UVB dose, indicating that the 18.24 kJ m^−2^ UVB dose negatively affected Rubisco and electron transport capacity of the photosynthetic apparatus. Variation in environmental conditions can modulate not only Rubisco activity but also its concentration^30^. In this study, our results showed that Rubisco content was not affected by the enhanced UVB (Supplementary Fig. S1), suggesting that a 18.24 kJ m^−2^ dose may negatively affect its activity. Similarly, the observed UVB-induced reduction of photosynthetic electron transport was also found in a previous study on *Chenopodium album* under strong UVB irradiance which damaged both electron acceptor and donor sides of PSII^31^.

In conclusion, the present study showed that quinoa regulates different mechanisms of response depending on the UVB irradiation dosage. Our results suggested that 1.69 W m^−2^ UVB radiation for 30 min doses induces UVB-specific signaling, which involves the interaction of several regulators in order to counteract ROS and avoid damage to PSII. On the other hand, 1.69 W m^−2^ UVB radiation for 60 min doses may promote UVB-independent pathways, where ROS overproduction was the predominant cause of tissue necrosis and probably inducing a transient repair mechanisms only during the first 3 DOT by activation of the pathogen-defense/wound-signaling pathways^32^. To our knowledge, there is no previous report that shows different responses of high-altitude Andean crops to regulate short acute UVB doses. Further studies to reveal the detailed mechanism developed by quinoa in response to different UVB doses are under progress.

## Materials and Methods

### Plant material and growth conditions

Seeds of quinoa (*Chenopodium quinoa* Willd.) variety réal were commercially obtained from Priméal (Peaugres, France). Seeds were surface sterilized with 1% (v/v) sodium hypochlorite solution and washed thoroughly with distilled water. Sterilized seeds were sown on MS^33^ medium containing 0.8% agar in a controlled growth chamber (12 h photoperiod, temperature 22 ± 1 °C, ambient relative humidity 75% and PPFD 100 µmol m^−2^ s^−1^). After 9 days, seedlings were transferred in plastic pots containing commercial soil and placed for other 23 days under the same growth conditions. Plants received distilled water three times a week and nutrient solution (NPK 5–5–5) once a week.

### UVB radiation treatments

Two climatic chambers were set to the following conditions: 12 h photoperiod, temperature 22 ± 1 °C, relative humidity 75% and PPFD 100 µmol m^−2^ s^−1^. One of the chambers was also equipped with three UVB-emitting lamps (Philips TL 20 W/01RS UVB Narrowband, Koninklijke Philips Electronics, Eindhoven, The Netherlands) with a peak emission at 311 nm. These lamps were mounted 45 cm above the tops of the plants. The spectra of the UV lamps were measured using a JAZ EL200-XR1 spectroradiometer (Ocean Optics, Dunedin, FL, USA). UV irradiances were measured using an UV-B sensor (SKU 430, Skye Instruments, Llandrindod Wells, UK). The spectral output of the UV tubes was weighted using the biological spectral weighting function described by Flint & Caldwell^34^. The calculated biologically effective daily UVB doses were 3.04 or 6.08 kJ m^−2^ for 30 min or 60 min 1.69 W m^−2^ UVB treatments, respectively. Pots of quinoa plants (32 days after sowing) were randomly distributed into the chambers and acclimated for 1 day. After acclimation, two different groups of plants were daily UVB irradiated for either 30 or 60 min (no consecutive experiments) at the middle of the photoperiod. The first treatment lasted 9 days and the second one 5 days. A third group of plants was maintained in the chamber without UVB. After the specified time points, first fully expanded leaves of UVB treated and untreated plants were collected and immediately frozen in liquid nitrogen and stored at −80 °C for the biochemical analyses. The photosynthetic measurements were performed immediately after UVB irradiation. Measurements of untreated plants did not show any significant differences over the time course study; therefore, only the time 0 was considered as a representative control (Supplementary Tables S1 and S2). For recovery test, a set of UVB treated and untreated plants were transferred to a controlled growth chamber (12 h photoperiod, temperature 22 ± 1 °C, ambient relative humidity 75% and PPFD 100 µmol m^−2^ s^−1^) for one week. The peak of UVB irradiance used in this work corresponds to a typical Mediterranean mid-altitude locality^35^.

### Chlorophyll Fluorescence

Chlorophyll fluorescence measurements were conducted on control and UVB treated quinoa leaves using a miniaturized pulse amplitude-modulated fluorometer (Mini-PAM; Heinz Walz GmbH, Effeltrich, Germany) as described by Huarancca Reyes *et al*.^36^. Briefly, the photon yield of PSII in the light (Φ_PSII_) was determined as Φ_PSII_ = (*F*_*m*_′ − *F*′)/*F*_*m*_′ at steady state, where *F*_*m*_′ is the maximum fluorescence yield with all PSII reaction centres in the reduced state obtained superimposing a saturating light flash during exposition to actinic light and *F*′ is the fluorescence at the actual state of PSII reaction centres during actinic illumination. The maximum PSII photochemical efficiency was evaluated in plants adapted to dark for at least 30 min as *F*_*v*_/*F*_*m*_ = (*F*_*m*_ − *F*_*o*_)/*F*_*m*_, where *F*_*o*_ and *F*_*m*_ are the minimal and the maximum fluorescence yield emitted by the leaves in the dark adapted state, respectively. Light response curves of Φ_PSII_ were carried out on fully expanded leaves over a range of PPFD between 25 and 1600 µmol m^−2^ s^−1^ as described by Scartazza *et al*.^37^.

### Gas exchange measurements

Leaf gas exchange parameters were measured on control and UVB treated quinoa plants using the LI-6400-40 portable photosynthesis system (Li-Cor, Lincoln, NE, USA). Instantaneous measurements of CO_2_ assimilation rate (*A*), stomatal conductance (*g*_*s*_) and intercellular CO_2_ concentration (*C*_*i*_) were performed on well-exposed and expanded leaves at CO_2_ concentration of 400 µmol mol^−1^, relative humidity ranging between 45–55%, leaf temperature of 25 °C and at both growing (100 µmol m^−2^ s^−1^) and saturating (1600 µmol m^−2^ s^−1^) PPFD. The *A*/*C*_*i*_ curves were carried out on fully expanded leaves over a range of CO_2_ concentration between 50 and 1800 μmol mol^−1^ at saturating PPFD values, using the method described by Scartazza *et al*.^37^. Each step comprised about 5 min for adjustment and stabilization of the gas exchange parameters. Values of maximal carboxylation rate (*V*_*cmax*_) and maximal light-driven electron transport rate (*J*_*max*_) were estimated by fitting the mechanistic model of CO_2_ assimilation to individual *A*/*C*_*i*_ response data^38^.

### Photosynthetic pigments analysis

Chlorophyll and carotenoids were extracted and analyzed as reported by Castagna *et al*.^39^. Carotenoids included β-carotene, neoxanthin, lutein, violaxanthin, antheraxanthin and zeaxanthin. Leaf disks were homogenized under dimmed room light in 100% HPLC-grade acetone with 1 mM sodium ascorbate. The extract was filtered through 0.2-µm filters (Sartorius Stedim Biotech, Goettingen, Germany) and analyzed by a Spectra System P4000 HPLC equipped with a UV 6000 LP photodiode array detector (Thermo Fisher Scientific, Waltham, MA) using a Zorbax ODS column (5 μm particle size, 250 × 4.6 mm Ø, Agilent Technologies, Santa Clara, CA, USA) at a flow rate of 1 mL min^−1^. Acetonitrile/methanol (85/15) and methanol/ethyl acetate (68/32) were used as solvent A and solvent B, respectively, according to the following gradient: solvent A: 100% (0–15 min), 100–0% (15–17.5 min), 0% (17.5–32 min), followed by 5 min re-equilibration in the initial condition before the next injection. Commercial standards of chlorophyll *a*, chlorophyll *b*, lutein and β-carotene were used for external calibration curves. Pigments were detected at 445 nm and quantified by injecting known amounts of pure standards (Sigma-Aldrich, Milan, Italy).

### Analysis of non-enzymatic antioxidant activities

Non-enzymatic antioxidant activities in the leaf tissues of quinoa plants were measured in terms of Trolox equivalent antioxidant capacity and total flavonoid content. Trolox equivalent antioxidant capacity was determined as described by Moles *et al*.^40^. Total flavonoids content was determined as described by Becatti *et al*.^41^ with minor modifications. Quinoa leaves (0.3 g) were extracted with 0.5 mL of 80% (v/v) methanol. Samples were then centrifuged at 10000 × *g* for 15 min at 4 °C, and the supernatant was used for flavonoids quantification. Amounts of 60 μL of 5% NaNO_2_, 40 μL of 10% AlCl_3_, and 400 μL of 1 M NaOH were added to 100 μL of liquid extract. The solution was diluted with 200 μL of distilled water and mixed. The content of flavonoids was determined by measuring the absorbance at 510 nm using a spectrophotometer. Results were calculated as mg of catechin equivalents.

### Protein Analysis

Total proteins were extracted and quantified as described by Pompeiano *et al*.^42^. Extracts were dissolved in SDS sample buffer (125 mM Tris-HCl, pH 6.8, 20% [v/v] glycerol, 4% [w/v] SDS, and 10% [v/v] β-mercaptoethanol) and analyzed by SDS-PAGE.

### Statistical analysis

Values presented are means ± standard error of three replicates. Following Bartlett’s test for homogeneity of variance, all data were subjected to analysis of variance (ANOVA) procedures as follows: data on Figs 1–7 were subjected at first to two-way ANOVA test to assess the effects of treatment (control, 30 min UVB and 60 min UVB) and time of treatment as well as their interaction on different tested parameters; data on Fig. 8 were subjected to a three-way ANOVA analysis taking into account PPFD level, treatment and time of treatment. Further one-way ANOVA analysis was performed as shown in Figs 1–8, and the mean values were compared by using Tukey test only when the previous ANOVA tests were significant for at least one of the above mentioned specific factors. For all parameters, the assumption of normality distribution was confirmed by the Shapiro-Wilks test. Data on Fig. 9 and Table 1 were only subjected to one-way ANOVA and Tukey tests to verify the effect of the treatments. Significant differences for all statistical tests were evaluated at the level of *P* < 0.05 using STATISTICA for Windows (Start-Soft, Inc., Tulsa, USA).

## Electronic supplementary material

Supplementary Information
