# Supplementary material for: Physiological effects of short acute UVB treatments in Chenopodium quinoa Willd
Source: Sci Rep. 2018 Jan 10;8:371. doi: 10.1038/s41598-017-18710-2 (PMC5762895; doi:10.1038/s41598-017-18710-2)
Supplement: Supplementary file 1 — Supplementary Information [file 41598_2017_18710_MOESM1_ESM.pdf]

***Scientific Reports* Supplementary Information**

Article title: Physiological effects of short acute UVB treatments in *Chenopodium quinoa* Willd.
Authors: Thais Huarancca Reyes, Andrea Scartazza, Antonella Castagna, Eric G. Cosio, Annamaria Ranieri & Lorenzo Guglielminetti

Supplementary Table S1. Measurement of different parameters in untreated plants at different times of the experimental course. Each value represents the mean ± standard error (n = 3). Significant differences between means within a row were tested using one-way ANOVA and Tukey tests ($P < 0.05$). ns, not significant. FW, fresh weight. V, Violaxanthin. A, Antheraxanthin. Z, Zeaxanthin.

	**0 d**	**5 d**	**9 d**
Φ_{PSII}	0.74 ± 0.01 ns	0.75 ± 0.01 ns	0.73 ± 0.01 ns
F_v/F_m	0.81 ± 0.00 ns	0.82 ± 0.01 ns	0.81 ± 0.00 ns
Chl *a* (mg g^{-1} FW)	2671 ± 110.7 ns	2526 ± 293.0 ns	2621 ± 270.8 ns
Chl *b* (mg g^{-1} FW)	689 ± 48.5 ns	706 ± 80.0 ns	619 ± 45.2 ns
Total Chl (mg g^{-1} FW)	3360 ± 157.5 ns	3232 ± 369.4 ns	3240 ± 316.0 ns
Carotenoids (mg g^{-1} FW)	607 ± 42.9 ns	597 ± 48.4 ns	551 ± 33.1 ns
Chl *a/b*	3.88 ± 0.15 ns	3.58 ± 0.13 ns	4.23 ± 0.22 ns
Carotenoids/Chl	0.18 ± 0.01 ns	0.19 ± 0.01 ns	0.17 ± 0.01 ns
Violaxanthin (mg g^{-1} FW)	146 ± 11.3 ns	179 ± 26.9 ns	150 ± 14.8 ns

Antheraxanthin ($mg\ g^{-1}$ FW)	6.15 ± 0.55 ns	5.34 ± 0.37 ns	8.07 ± 1.24 ns
Zeaxanthin ($mg\ g^{-1}$ FW)	3.61 ± 0.26 ns	3.82 ± 0.55 ns	3.32 ± 0.51 ns
Violaxanthin/Chl	0.04 ± 0.00 ns	0.06 ± 0.01 ns	0.05 ± 0.00 ns
Antheraxanthin/Chl	0.002 ± 0.00 ns	0.002 ± 0.00 ns	0.002 ± 0.00 ns
Zeaxanthin/Chl	0.001 ± 0.00 ns	0.001 ± 0.00 ns	0.001 ± 0.00 ns
[(0.5A+Z)/(V+A+Z)] ×100%	4.43 ± 0.51 ns	3.56 ± 0.57 ns	4.53 ± 0.33 ns
Flavonoids ($mg\ g^{-1}$ FW)	0.90 ± 0.04 ns	0.84 ± 0.06 ns	0.88 ± 0.03 ns
Antioxidants (nmoles trolox g^{-1} FW)	1997 ± 41.8 ns	2017 ± 73.4 ns	1961 ± 62.4 ns

Supplementary Table S2. Measurement of different parameters in untreated plants at different times of the experimental course. Each value represents the mean ± standard error (n = 3). Significant differences between means within a row were tested using one-way ANOVA and Tukey tests ($P < 0.05$). ns, not significant. A, CO_2 assimilation rate. g_s, stomatal conductance. C_i, intercellular CO_2 concentration.

	**0 d**	**1 d**	**3 d**
**PPFD 100 µmol m^{-2} s^{-1}**			
A (µmol m^{-2} s^{-1})	3.09 ± 0.67 ns	3.01 ± 0.48 ns	3.17 ± 0.54 ns
g_s (mol m^{-2} s^{-1})	0.17 ± 0.03 ns	0.18 ± 0.02 ns	0.16 ± 0.03 ns
C_i (µmol mol^{-1})	359.4 ± 3.6 ns	336.3 ± 6.9 ns	348.9 ± 12.7 ns
**PPFD 1600 µmol m^{-2} s^{-1}**			
A (µmol m^{-2} s^{-1})	16.82 ± 1.38 ns	17.56 ± 1.58 ns	16.69 ± 0.97 ns
g_s (mol m^{-2} s^{-1})	0.27 ± 0.04 ns	0.25 ± 0.05 ns	0.28 ± 0.03 ns
C_i (µmol mol^{-1})	273.2 ± 11.2 ns	286.5 ± 8.3 ns	265 ± 12.5 ns

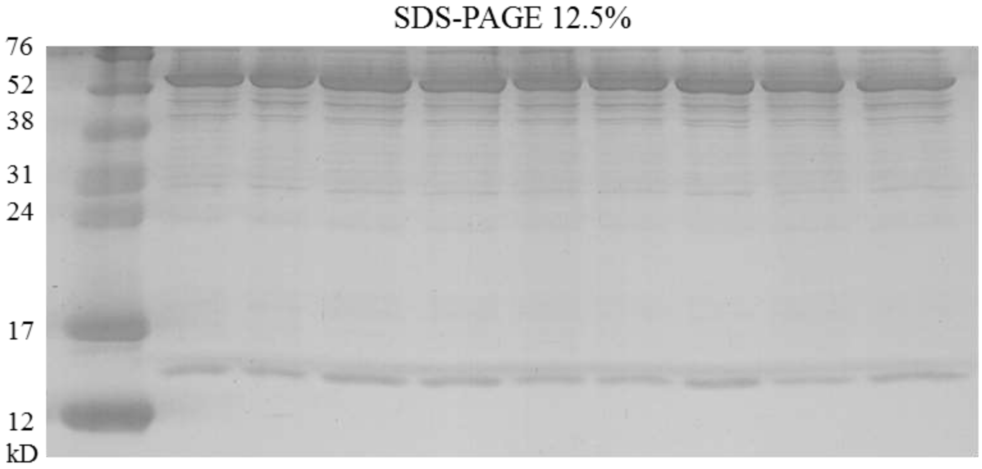

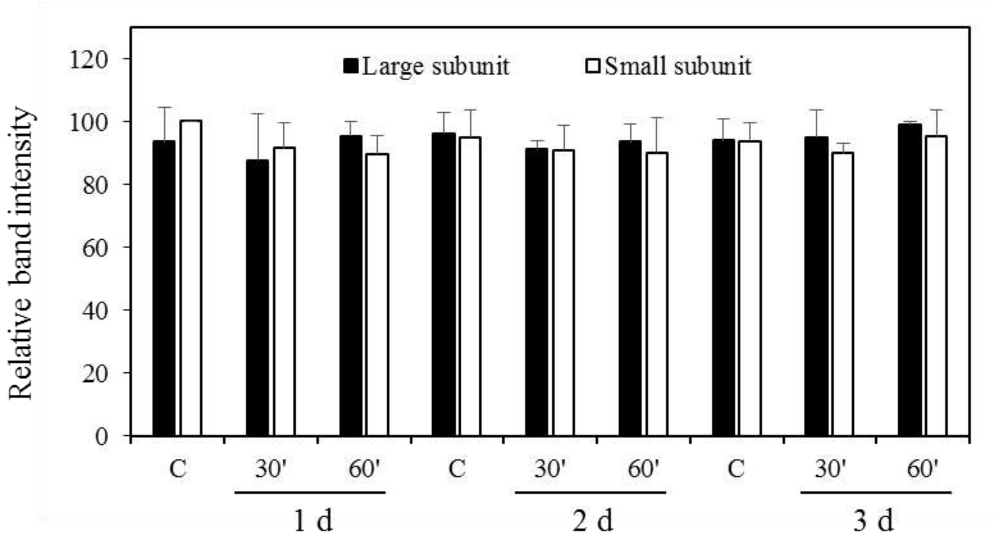

Supplementary Figure S1. Effect of different UVB doses on Rubisco content of quinoa. Protein extraction was performed using leaves from control and 1, 2, 3 days UVB treated plants. Quinoa plants were exposed to 100 µmol m^{-2} s^{-1} photosynthetic photon flux density (PPFD) and 1.69 W m^{-2} UVB. UVB were daily applied for 30 or 60 min. Control plants only received PPFD. A representative SDS-PAGE is shown above the Rubisco subunits quantification. The positions of the molecular weight markers (kD) are indicated on the left of the gel. Gel images were generated by using an Epson scanner XP332 with its respective software set at default (EPSON Scan). Original gel images were used for quantification and figure design. Band intensities from each Rubisco subunit were quantified by the use of ImageJ software. The densitometric analysis is reported as histogram, in which the more intense Rubisco band was considered 100% arbitrary

protein amount. Data are the mean of three replicates (±SD). C, control.
